# The Bombyx mori Nucleopolyhedrovirus GP64 Retains the Transmembrane Helix of Signal Peptide to Contribute to Secretion across the Cytomembrane

**DOI:** 10.1128/spectrum.01913-22

**Published:** 2022-08-08

**Authors:** Bifang Hao, Lin Liu, Na Liu, Luping Sun, Fengxiu Fan, Jinshan Huang

**Affiliations:** a Jiangsu Key Laboratory of Sericultural Biology and Biotechnology, School of Biotechnology, Jiangsu University of Science and Technology, Zhenjiang, Jiangsu, People’s Republic of China; b Key Laboratory of Genetic Improvement of Sericulture in the Ministry of Agriculture, Sericultural Research Institute, Chinese Academy of Agricultural Science, Zhenjiang, Jiangsu, People’s Republic of China; Oklahoma State University, College of Veterinary Medicine

**Keywords:** fusion protein, baculovirus, signal peptide, BmNPV, secondary structure

## Abstract

Bombyx mori nucleopolyhedrovirus (BmNPV) is the primary pathogen of silkworms that causes severe economic losses in sericulture. GP64 is the key membrane fusion protein that mediates budded virus (BV) fusion with the host cell membrane. Previously, we found that the n-region of the GP64 signal peptide (SP) is required for protein secretion and viral pathogenicity; however, our understanding of BmNPV GP64 remains limited. Here, we first reported that BmNPV GP64 retained its SP in the mature protein and virion in only host cells but did not retain in nonhost cells. Uncleaved SP mediates protein targeting to the cytomembrane or secretion in Bombyx mori cells. The exitance of the n-region extended the transmembrane helix length, which resulted in the cleavage site to be located in the helix structure and thus blocked cleavage from signal peptidase (SPase). Without the n-region, the protein fails to be transported to the cytomembrane, but this failure can be rescued by the cleavage site mutation of SP. Helix-breaking mutations in SP abolished protein targeting to the cytomembrane and secretion. Our results revealed a previously unrecognized mechanism by which SP of membrane fusion not only determines protein localization but also determines viral pathogenicity, which highlights the escape mechanism of SP from the cleavage by SPase.

**IMPORTANCE** BmNPV is the primary pathogen of silkworms, which causes severe economic losses in sericulture. BmNPV and Autographa californica multiple nucleopolyhedrovirus (AcMNPV) are closely related group I alphabaculoviruses, but they exhibit nonoverlapping host specificity. Recent studies suppose that GP64 is a determinant of host range, while knowledge remains limited. In this study, we revealed that BmNPV GP64 retained its SP in host cells but not in nonhost cells, and the SP retention is required for GP64 secretion across the cytomembrane. This is the first report that a type I membrane fusion protein retained its SP in mature proteins and virions. Our results unveil the mechanism by which SP GP64 escapes cleavage and the role of SP in protein targeting. This study will help elucidate an important mechanistic understanding of BmNPV infection and host range specificity.

## INTRODUCTION

Baculoviruses are a family of enveloped DNA viruses that mainly infect insects from the orders Lepidoptera, Diptera, and Hymenoptera and have important applications in the areas of insect pesticides, protein expression, and gene therapy (1). Bombyx mori nucleopolyhedrovirus (BmNPV) is a primary silkworm pathogen that causes major economic losses in sericulture, and no efficient method is available to prevent virus infection. Autographa californica multiple nucleopolyhedrovirus (AcMNPV) is a well-studied group I alphabaculovirus with high identity to BmNPV (2), but exhibits nonoverlapping host specificity (2, 3). AcMNPV infects a much more diverse set of insects and insect cell lines than BmNPV (3). For example, AcMNPV replicates in Spodoptera frugiperda (Sf) cells but not BmN cells; conversely, BmNPV replicates in BmN cells but not Sf cells. Several genes, such as *DNA helicase* (4, 5) and host cell-specific factor-1 (6, 7), were reported as host range factor determinants. GP64 was also recently suggested to be a determinant of the BmNPV and AcMNPV host range (1, 3, 8), while the mechanism remains largely unknown.

GP64 is the key membrane fusion protein (MFP) that mediates baculovirus budded virus (BV) membrane fusion with the host cell membrane by conformational change. GP64 is a type I integral membrane protein (IMP) that is targeted to the cytomembrane by its signal peptides (SP), leading to the secretory pathway. Typical SP shares tripartite structural features: a positively charged n-region, a most essential hydrophobic region (h-region), and a c-region located near the cleavage site of SP (9–11). SP can be removed co or posttranslationally by the cellular membrane-bound signal peptidase (SPase) complex (12). Only a few SPs in mature lipoproteins are not cleaved due to the lack of a functional SPase cleavage site (13, 14). Viral membrane proteins have n-regions of SPs that vary tremendously in length and amino acid composition (15). Most SPs that contain a typical n-region with 0 to 2 basic residues are removed and degraded after translocation (16), while the cleavage of a few viral SP does not occur until after protein folding and/or modification (17–19). Several viral glycoprotein SPs with a longer n-region are inserted into the membrane to form a protein complex that is involved in viral infection (20–22).

Notably, a controversial problem for GP64 is the initiation site. Two initiation codons were found in frame with the GP64 ORF in the database, and these two sites were used for different baculovirus GP64 annotations. Chang and Blissard demonstrated that AcMNPV GP64 translated from both sites, while the main product of GP64 was initiated from the second start codon (23). The alternative translation initiation at in-frame AUG codons generated functionally distinct isoform proteins (24) or subcellular location differentiated proteins (25). GP64 translated from the second initial sites resulted in the absence of the SP n-region; we recently found that the n-region of BmNPV GP64 was required for heterogeneous protein cytomembrane localization (26). Moreover, GP64 with full-length SP showed stronger virulence and a special dependence on cholesterol than a virus harboring GP64 without an n-region (27). Bioinformatic prediction indicated that SP was not cleaved for the n-region; therefore, whether BmNPV GP64 retained its SP in the virion needs to be clarified.

In this study, we reported that BmNPV GP64 retained its SP in the mature virion in host cells but not in nonhost cells. This uncleaved SP was required for BmNPV GP64 cytomembrane localization in the host cell. The absence of the n-region caused SP cleavage and protein intracellular localization. The uncleaved helix is required for protein secretion across the cytomembrane, and helix-breaking mutation abolishes protein transport across the cytomembrane. To our knowledge, this is the first report that type I IMP retained its SP although it contained an SPase cleavage site, which will facilitate our understanding of MFP function and evolution.

## RESULTS

### Bioinformatic analysis showed that the cleavage of GP64 SP varied in viruses.

**FIG 1:**
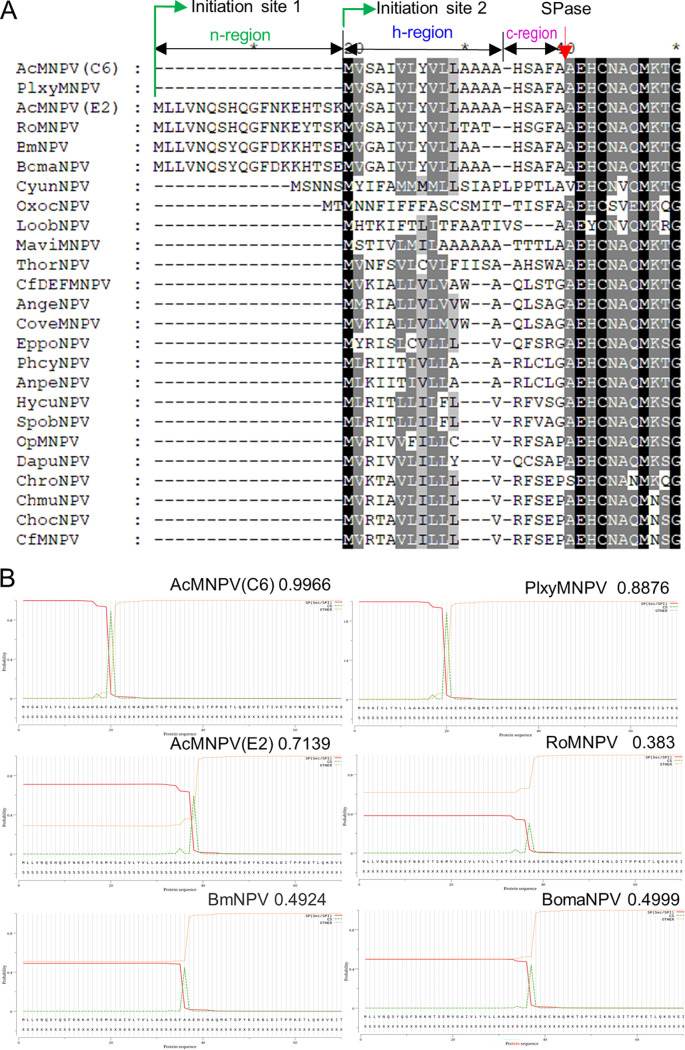
SP composition of alphabaculovirus GP64 and cleavage prediction. (A) Composition and alignment of the GP64 N-terminus of group I alphabaculoviruses by MegAlign (6.0); AcMNPV (C6) (AAA66758), PlxyMNPV (ABE68511), AcMNPV (E2) (AIU56980), RoMNPV (AAN28029), BmNPV (AVP27270), BomaNPV (YP_002884350), CyunNPV (YP_010086628), OxocNPV (YP_009666553), LoobNPV (YP_009666390), MaviMNPV (YP_950827), ThorNPV (YP_007250533), CfDEFMNPV (NP_932732), AngeNPV (AAS83210), CoveMNPV (YP_009118511), EppoNPV (NP_203281), PhcyNPV (AFY62837), AnpeNPV (YP_611000), HycuNPV (YP_473216), SpobNPV (AUR45058), OpMNPV (NP_046282), DapuNPV (AKR14107), ChroNPV (YP_008378382), ChmuNPV (YP_008992122), ChocNPV (AGR56918), CfMNPV (AAA67522). The green arrows show the differential initiation sites. The red arrow shows the cleavage site. (B) Cleavage prediction of selected alphabaculoviruses GP64 by SignalP 5.0. The probability values of each virus GP64 are shown in the figures.

**FIG 2:**
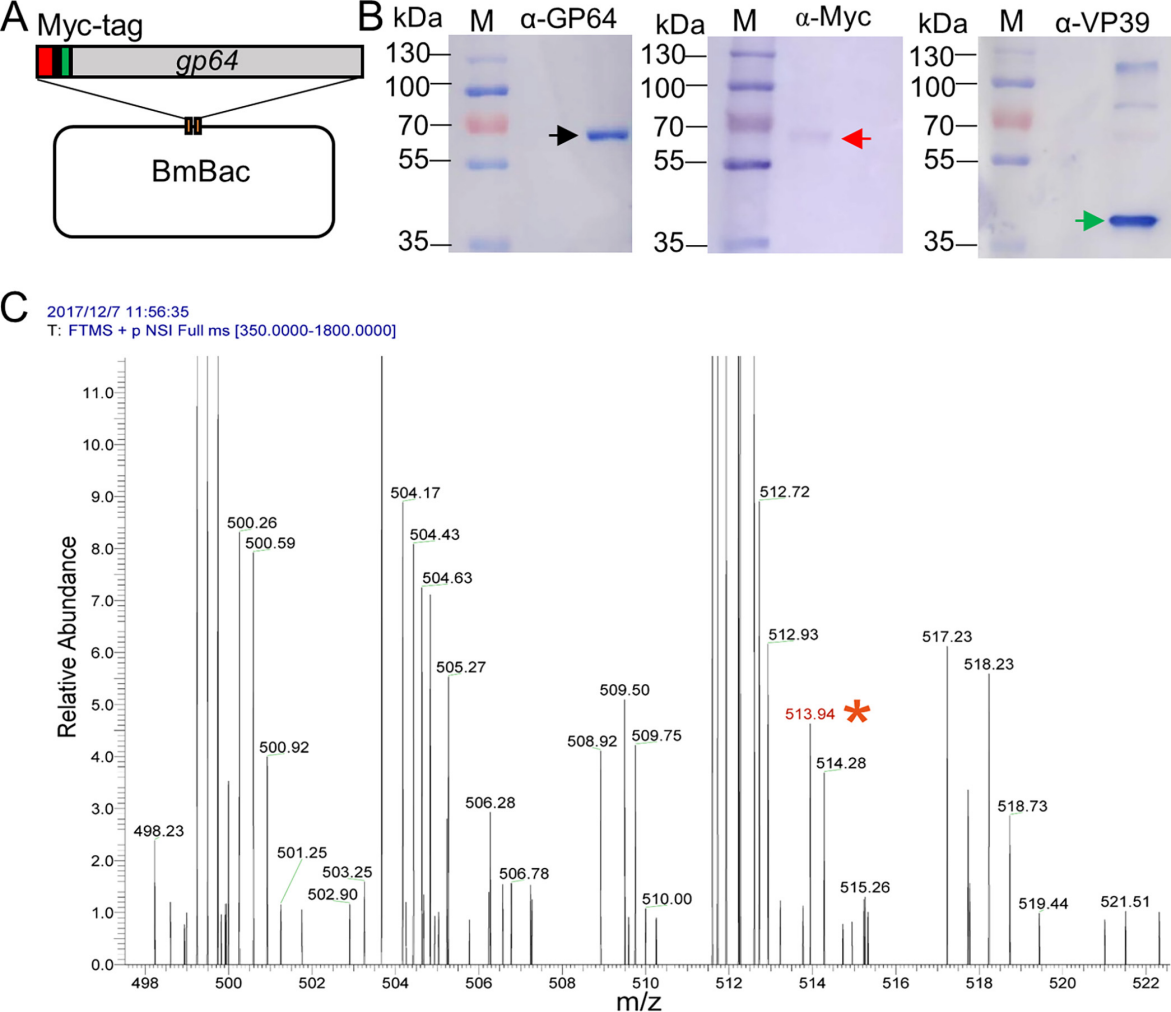
Cleavage analysis of BmNPV GP64 SP in host cells. (A) Construction of the Myc-tagged GP64 recombinant BmNPV bacmid. The gray box shows the GP64-encoding region; the red and green boxes represent the n-region and h-/c-region of GP64, respectively; and the Myc tag is shown by the black line. (B) Western blot assay of mature BVs incorporated with Myc-tagged GP64. BVs were collected and purified by sucrose gradient ultracentrifugation; then, the BVs were subjected to Western blot analysis with antibodies against GP64, Myc, and VP39. The black arrow shows the GP64 reaction band, the red arrow shows the immunoreaction band to the Myc-specific antibody, and the VP39 band is shown by the green arrow. (C) Peptide profile identified by MS from BV membrane proteins. WT BVs were purified by ultracentrifugation, the membrane proteins of BVs were separated, and the GP64 band was subjected to MS analysis. The red asterisk shows the peptide fragment (LLVNQSYQGFDKK), which is the n-region sequence of GP64 SP.

To investigate SP cleavage, 25 alphabaculovirus GP64s were aligned and the N-terminus of GP64 was shown in Fig. 1A. GP64 shared a high amino acid sequence identity, while several variations were found in the SP region of GP64. There were two initiation AUG codons in frame with GP64 ORFs, and the alternative initiation AUG codons generated two types of GP64s (Fig. 1A). The second codon was used for the annotation of GP64 in AcMNPV (C6), Plutella xylostella multiple nucleopolyhedrovirus (PlxyMNPV), and other alphabaculoviruses, which generated GP64 without the n-region. However, the first in-frame ATG is used for GP64 annotation in AcMNPV (E2 strain), BmNPV, Rachiplusia ou multiple nucleopolyhedrovirus (RoMNPV), and Bombyx mandarina nucleopolyhedrovirus (BomaNPV), which generated GP64 with a typical SP. Without the n-region, SP was removed from GP64 with high probability by SignalP5.0 prediction (28) (Fig. 2B), while the presence of the n-region caused noncleavage of SP except for AcMNPV(E2); the cleavage probability was 0.4924, 0.4999, and 0.383 in BmNPV, BomaNPV, and RoMNPV, respectively. Thus, whether baculovirus GP64 contained an uncleaved SP needs to be clarified.

### BmNPV GP64 retained its SP in mature virions.

To clarify the noncleavage of SP, BmNPV GP64 was selected for subsequent study. First, a recombinant BmNPV bacmid with Myc-tagged GP64 (BmBac-Myc-gp64) was constructed, in which the Myc-tag was inserted into the GP64 SP between the n-region and h-region (positions 18 and 19) (Fig. 2A). The recombinant bamids were transfected into BmN cells, and the BVs were propagated by infection of BmN cells. Then, the recombinant BVs were collected by sucrose gradient ultracentrifugation and subjected to Western blot analysis. To our surprise, although weak, a specific band was detected using Myc-antibody (Fig. 2B, red arrow), which was also detected by GP64-antibody (Fig. 2B, black arrow), and VP39 served as a control (Fig. 2B, green arrow). If SP was removed in the GP64 translocation process, the Myc-tag should not be detected in BV. This result indicated that Myc-tagged SP was not cleaved from mature GP64, and this chimeric GP64 was assembled into mature BVs in BmN cells. However, whether the Myc insertion in the SP interfered with cleavage was ambiguous. Second, wild-type BVs were collected by ultracentrifugation, the BV membrane proteins were separated, and the GP64 band was subjected to MS analysis. A peptide fragment (LLVNQSYQGFDKK), the exact n-region sequence of the GP64 SP, was identified (Fig. 2C, red asterisk), which confirmed that noncleavage of the Myc-tagged SP did not result from the insertion of the Myc-tag. These results indicated that BmNPV GP64 SP was not cleaved in host cells even though it contained a cleavage site.

### The noncleavage of BmNPV GP64 SP occurred in BmN cells but not in Sf9 cells.

**FIG 3:**
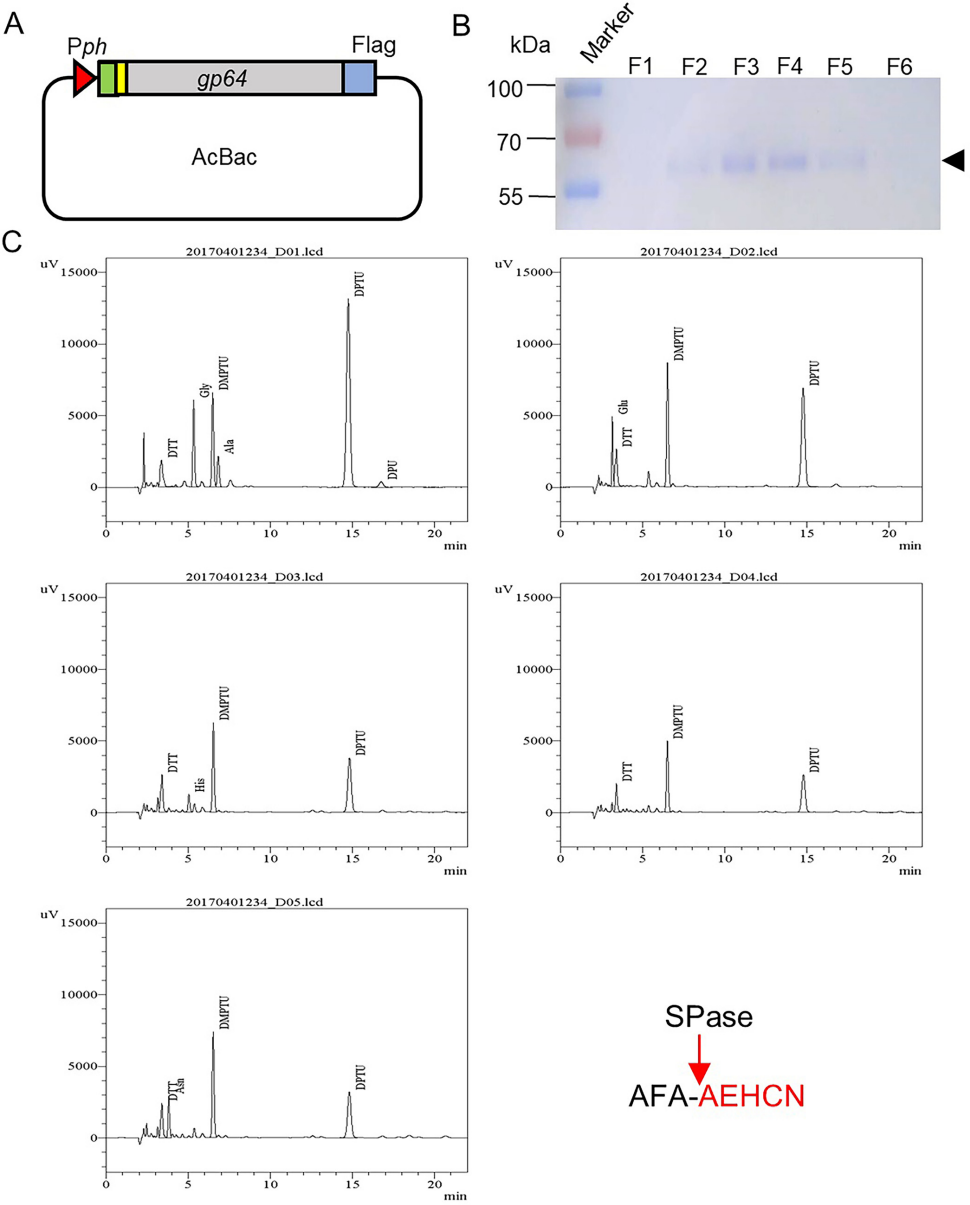
Cleavage analysis of BmNPV GP64 SP in nonhost cells. (A) BmNPV GP64 expression by Acbacmid in Sf9 cells. Full-length BmNPV GP64 was driven under the promoter of ph (Pph), the gray box presents the gp64 gene without the TMD, and the yellow box shows the Flag-tag. (B) SDS–PAGE analysis of purified protein. The recombinant protein was purified from the media with a Flag affinity column. F1 to F6: fractions of the elution; the arrow shows the fusion protein GP64-Flag. (C) Cleavage analysis of GP64-Flag by N-terminal sequencing. The graphs showed the five amino acids (AEHCN) of the N terminus of GP64-Flag through protein N-terminal sequencing analysis. The red letters show the exact N-terminal amino acid sequence, and the red arrow shows the SPase cleavage site.

To clarify whether the noncleavage of SP was host specific, BmNPV full-length (FL) GP64 without a transmembrane domain (TMD) was expressed by the AcMNPV bacmid in nonhost cells (Sf9 cells), and a Flag-tag was fused to the C-terminus of GP64 for purification (Fig. 3A). The recombinant bacmid was transfected and infected into Sf9 cells for GP64-Flag expression on a large scale, and a single band was purified in the elution by SDS–PAGE (Fig. 3B). This elution was concentrated and then subjected to protein N-terminal sequencing. The results showed that the N-terminal sequence was AEHCN, which is the exact sequence of GP64 after the cleavage site (Fig. 3C), which indicated that BmNPV GP64 SP was removed in nonhost cells.

### The n-region of SP is required for protein secretion in host cells but not in nonhost cells.

**FIG 4:**
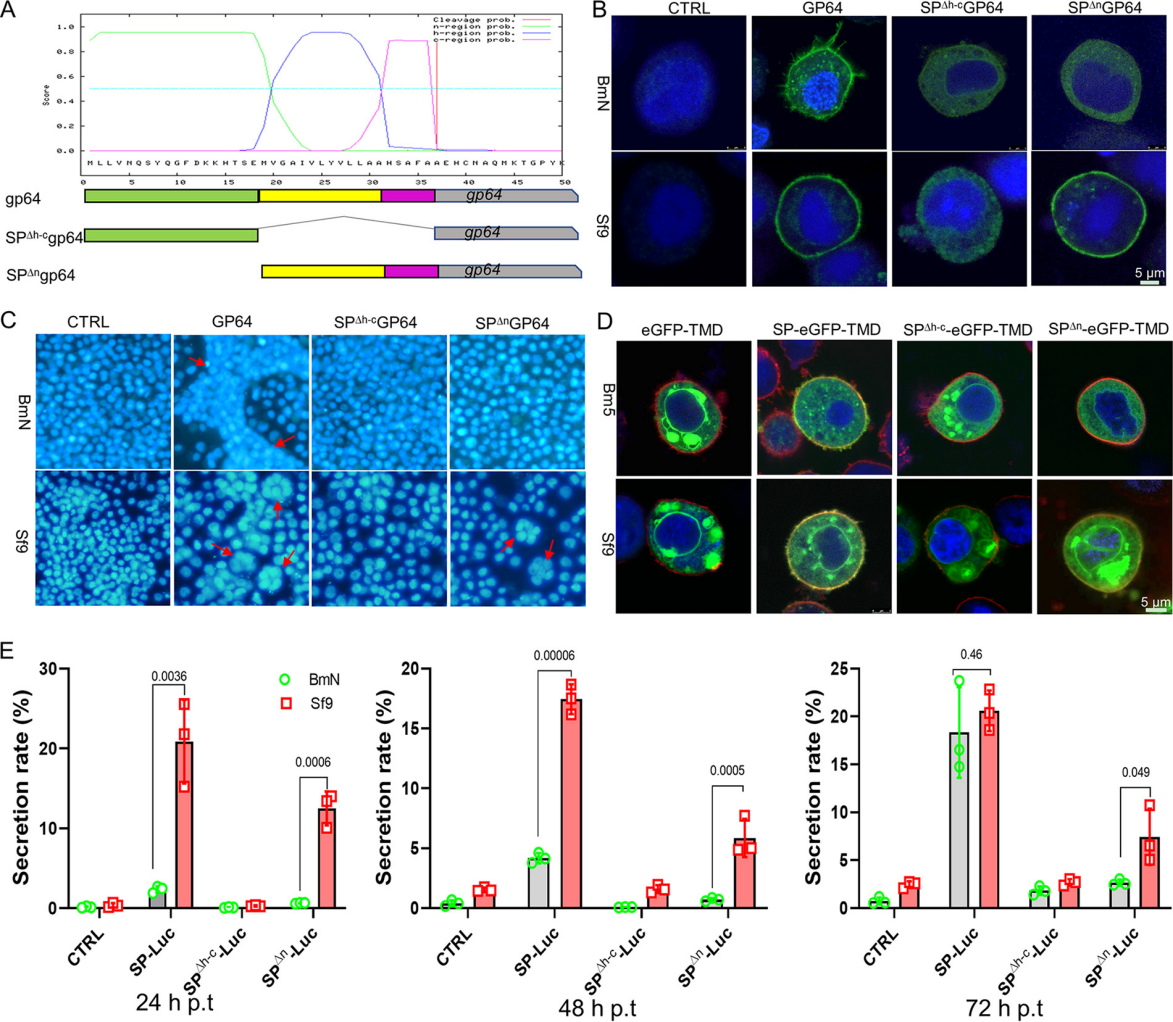
The N-region of SP mediates protein secretion in different cells. (A) Schematic diagram of transient expression vectors of GP64. A hydropathy plot of GP64 SP is shown in colored boxes; green represents the n-region, yellow represents the h-region, and magenta represents the c-region. (B) Localization of GP64 with three different SPs. BmN or Sf9 cells were transfected with pIZ/V5, pIZ/V5-gp64, pIZ/V5-SP^Δh-c^gp64, or pIZ/V5-SP^Δn^gp64. Then, the cells were fixed and prepared for immunofluorescence assays with a GP64 antibody and a FITC-conjugated secondary antibody at 72 h.p.t., and DNA was stained with Hoechst. Bar, 5 μm. (C) Membrane fusion activity assay. Transfected BmN and Sf9 cells in D were exposed to low-pH TC100 medium (pH 4.5) to induce membrane fusion at 72 h.p.t. for 5 min, the cells were stained with Hoechst 33258 for 30 min, and the images were recorded at 4 h posttriggering. Red arrows show the syncytia (more than six nuclei). (D) Localization assay of eGFP with a different region of SP. FL-SP or a partial region of SP was linked with egfp-TMD in the transient expression vectors, and then Bm5 and Sf9 cells were transfected with these constructs. The membrane and nucleus were stained with R18 and Hoechst at 72 h.p.t., respectively. Bar, 5 μm. (E) Comparison of the secretion efficiency of luciferase led with the SPs. The transient expression vector of the *luciferase* gene led with different SPs was transfected into BmN or Sf9 cells. The media and the cells were harvested and lysed for luciferase activity assays at different time points posttransfection. The secretion rate was calculated with RLU in the medium averaged by the total RLU. All experiments included three independent repeats, and *P* values were generated with a two-tailed *P* value from a *t* test in GraphPad Prism 8.

As the viral MFP, GP64 is transported to the cytomembrane and is assembled into BVs in the budding process. Therefore, we next explored the role of different regions in GP64 localization. GP64 with an FL-SP or a partial region of the SP (SP^Δh-c^GP64 and SP^Δn^GP64) was inserted into transient expression vectors (Fig. 4A). Then, BmN or Sf9 cells were transfected with these vectors, and GP64 localization was detected by immunofluorescence at 72 h posttransfection (h.p.t.). As shown in Fig. 4B, in BmN cells, no fluorescence was observed in the control (pIZ/V5) (Fig. 4B, CTRL panel), and a clear fluorescence ring was displayed around the cells in GP64-transfected cells, which indicated that FL-SP mediated GP64 targeting on the cytomembrane. However, no fluorescence was observed on the cytomembrane in the SP^Δh-c^GP64 or SP^Δn^GP64 treatments, and the fluorescence was distributed in the cytoplasm (Fig. 4B, upper panel). However, in Sf9 cells, SP^Δh-c^GP64 was localized in the cytoplasm, while SP^Δn^GP64 was localized to the cytomembrane, which was similar to GP64 localization to the plasma membrane of Sf9 cells (Fig. 4B, lower panel). Furthermore, the membrane fusion analysis was in accord with those of the protein localization assay. Syncytia were formed in BmN cells with GP64 expression, and no syncytia were formed in SP^Δh-c^GP64 and SP^Δn^GP64 cells (Fig. 4C, upper panel). In Sf9 cells, no syncytia were formed in SP^Δh-c^GP64, and syncytia were observed in GP64- or SP^Δn^GP64-expressing cells (Fig. 4C, lower panel). The fusion result was in accord with that of the localization assay.

The leader function of SPs was further analyzed by heterologous gene transient expression. The same results were obtained in the eGFP-TMD localization assay in another host cells (Bm5) and nonhost cells (Sf9) (Fig. 4D), which further verified that the n-region was required for protein localization in host cells. Quantitative analysis was performed by Luciferase secretion comparison. The SP-Luc secretion rate increased slowly over time in BmN cells (Fig. 4E); however, SP-Luc showed a stable secretion rate (~20%) in Sf9 cells, and no significant difference was detected at 72 h.p.t. with that in BmN cells. SP^Δn^-Luc shared a certain secretion in Sf9 cells, but it showed almost no secretion in BmN cells at 24 and 48 h.p.t., and an increased secretion rate was found at 72 h.p.t., which may be caused by the leakage of the transfected cells for the long-term incubation because the secretion rate of CTRL (Luc) and SP^Δh-c^-Luc was increased at a late stage (Fig. 4E). Taken together, these findings confirmed that the n-region was required for protein secretion in host, BmN cells but not in nonhost, Sf9 cells.

### Protein secretion was dependent on the n-region length but not key residues in the n-region.

**FIG 5:**
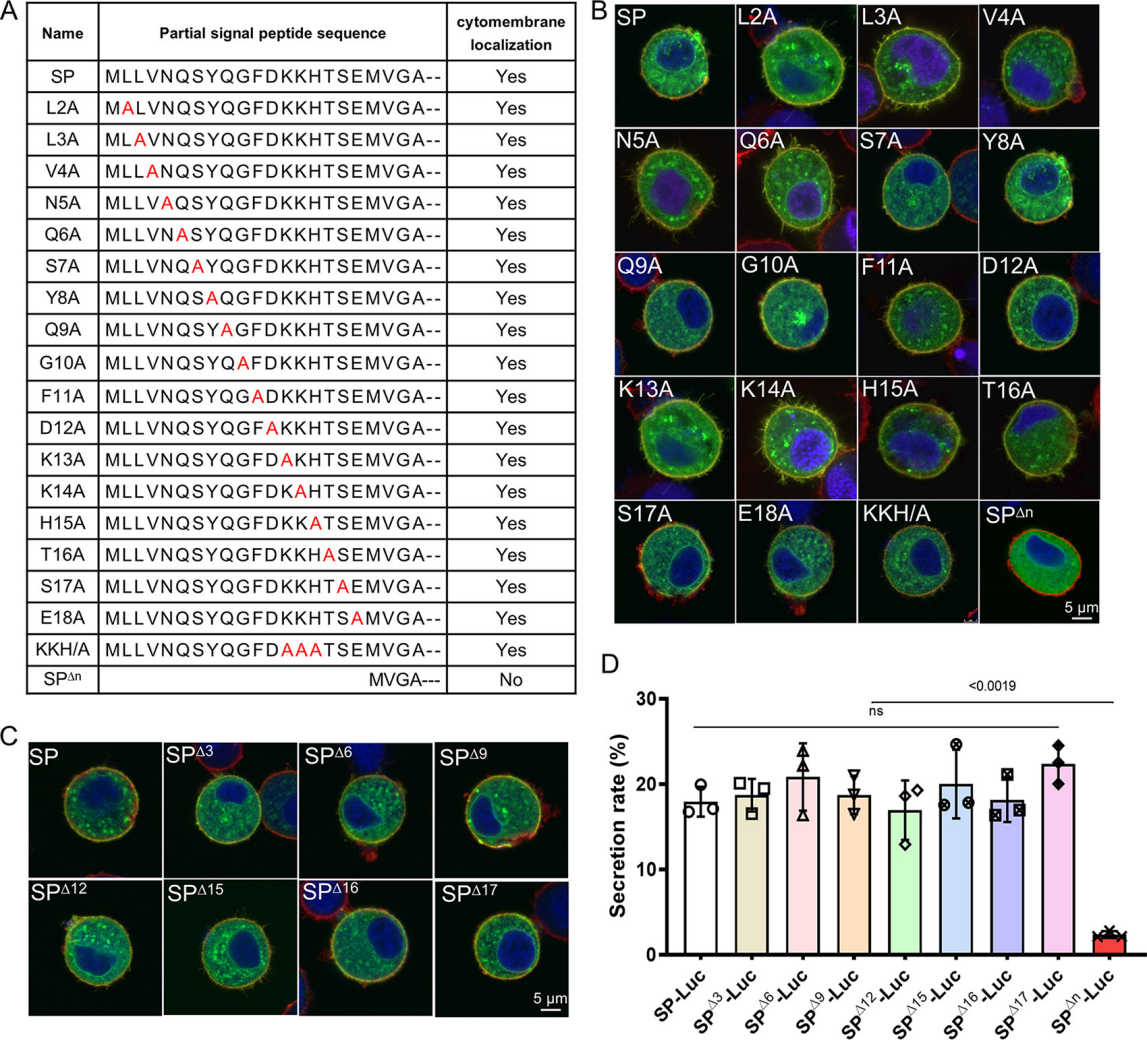
Mutagenesis and truncation assay on the n-region of SP. (A) Alanine mutagenesis scanning of amino acids in the n-region and localization assay in BmN cells. Point-mutated SPs were synthesized and inserted into the pIZ/V5 vector expressing eGFP-TMD. Then, BmN cells were transfected with the mutants and stained with R18 and Hoechst at 72 h.p.t., and localization was recorded by laser confocal microscopy. Yes indicates cytomembrane localization. (B) Localization assay of eGFP with alanine mutagenesis SP in BmN cells. BmN cells were transfected with a series of point-mutated constructs, and the transfected cells were imaged at 72 h.p.t. Bar, 5 μm. (C) Localization assay of eGFP with n-region truncated SPs in BmN cells. BmN cells were transfected with a series of truncated constructs, and the transfected cells were imaged at 72 h.p.t. Bar, 5 μm. (D) Comparison of luciferase secretion with the truncated n-region. The *luciferase* gene was inserted into the pIZ/V5 vector with the truncated n-region of SP. Then, BmN cells were transfected with these vectors, and the supernatant and cells were harvested at 72 h.p.t. Luciferase activity was determined and analyzed. The secretion rate was calculated with RLU in the medium averaged by the total in the cell lysate and the medium. All experiments included three independent repeats, and *P* values were generated with a two-tailed *P* value from Student’s *t* test.

To clarify why the n-region was vital for protein secretion in host cells, the amino acids in the n-region were scanned by alanine mutagenesis analysis with the transient expression vector SP-eGFP-TMD; however, all the mutants had the same localization as that of SP-eGFP-TMD, as each colocalized on the cytomembrane (Fig. 5A and B). This indicated that no key amino acid in the n-region was essential for protein secretion. Because the length of the n-region influences SPase processing (29, 30), we next analyzed whether n-region truncation altered protein secretion. A series of truncated SPs linked with eGFP-TMD or *luciferase* was constructed, and the localization and secretion rate were checked at 72 h.p.t. However, the truncated n-region did not alter eGFP localization (Fig. 5C), and the truncated constructs shared a secretion rate similar to that of SP-Luc (Fig. 5D). No significant difference was detected between these truncated and wild-type constructs, whereas significant differences were detected between the truncated constructs and SP^Δn^-Luc. Together, these results indicated that the n-region containing over one residue can mediate protein secretion.

### The uncleaved hydrophobic helix is required for protein secretion in BmN cells.

**FIG 6:**
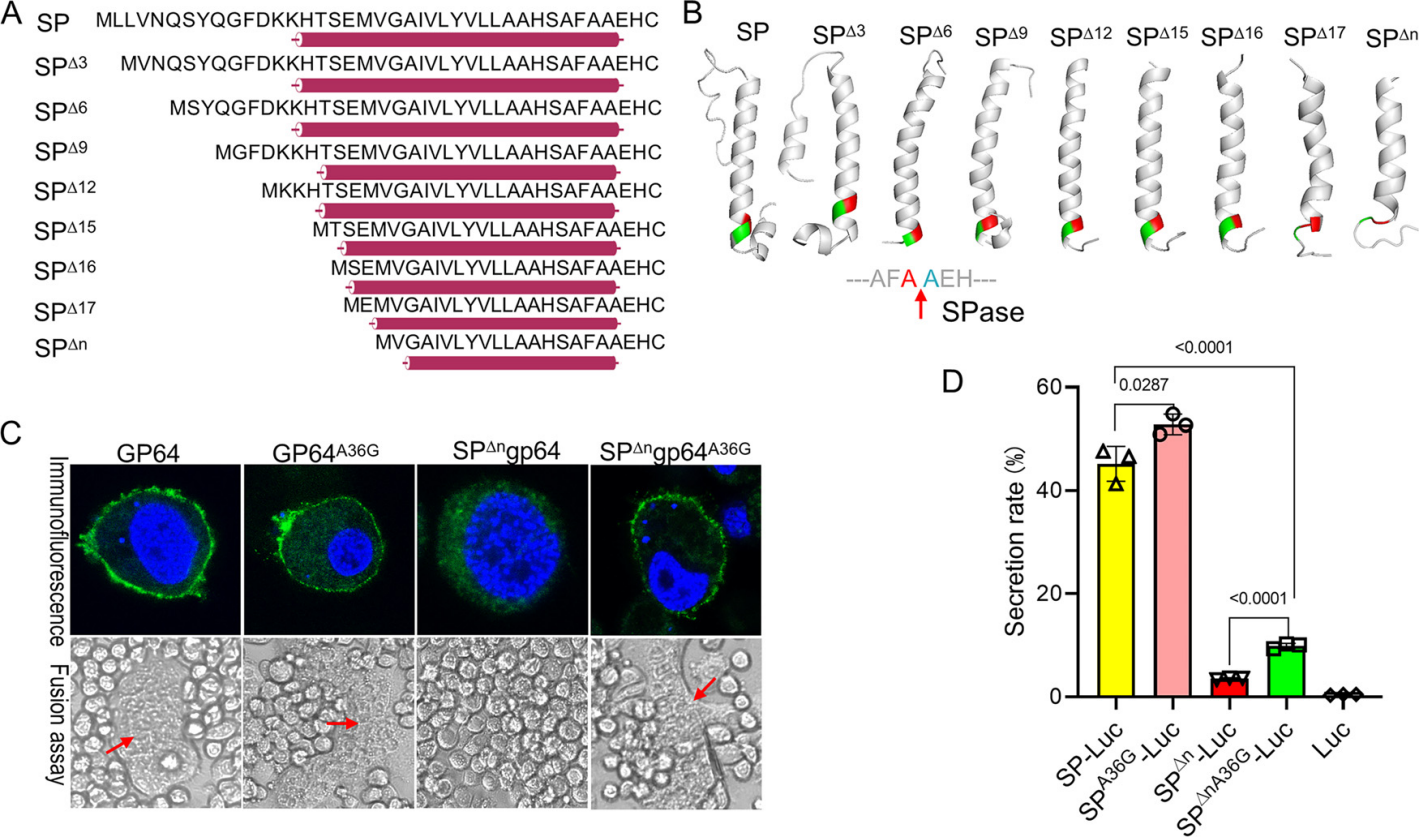
Verification of the transmembrane helix in protein secretion. (A) Schematic diagram and prediction of the secondary structure of the truncated n-region around the cleavage site by JPred. The purple column shows the α-helix structure. (B) Secondary structure analysis of SPs truncated by Phyre2. The cleavage site residues are shown in red and green letters, and the arrow shows the SPase cleavage site. (C) Localization and membrane fusion assay of GP64 mutations on the SPase recognition site. The key residue of the cleavage site alanine of SP was mutated to glycine. BmN cells were transfected with pIZ/V5-gp64, pIZ/V5-gp64^A36G^, pIZ/V5-SP^Δn^gp64, and pIZ/V5-SP^Δn^gp64^A36G^, and the cells were then fixed at 72 h.p.t. Immunofluorescence was observed with an anti-GP64 antibody and a FITC-conjugated secondary antibody, and DNA was stained with Hoechst. Meanwhile, the transfected BmN cells were exposed to low-pH TC100 medium (pH 4.5) to induce membrane fusion at 72 h.p.t. The images were recorded at 4 h postinduction, and red arrows show the syncytia. (D) Comparison of luciferase secretion with the cleavage site mutated SP. BmN cells were transfected with pIZ/V5-SP-Luc, pIZ/V5-SP^Δn^-Luc, pIZ/V5-SP^A36G^-Luc, and pIZ/V5-SP^ΔnA36G^-Luc, and the luciferase secretion rate and significance were analyzed at 72 h p.t.

The transmembrane segment of the membrane protein is predicted to form a helix (31), and this secondary structure around SP blocks proteolysis from SPase (19). Then, the secondary structure of truncated SPs was first predicted by JPred. The results showed that the n-region extended the length of the α-helix, which covered the whole h-region and c-region (Fig. 6A, purple bars). The secondary structure was also predicted by Phyre2 and analyzed by PyMOL (DeLano Scientific LLC), which further revealed that the helix length decreased accordingly in the truncated SPs (Fig. 6B). Notably, both cleavage sites were covered by the long helix (Fig. 6B, shown in red and green). When the n-region was deleted completely, one residue of the cleavage site was located in the helix, and two residues were exposed in the loose loop (Fig. 6B, SP^Δn^); therefore, we suggested that the cleavage site was recognized and cleaved by SPase, which resulted in failure of secretion.

To verify this hypothesis, the SPase recognition site AFA was mutated to AFG which will prevent cleavage, and the expression of GP64^A36G^ and SP^Δn^GP64^A36G^ was checked in BmN cells by transient expression. As expected, GP64^A36G^ was efficiently expressed and secreted into the BmN cell cytomembrane (Fig. 6C), and fusion activity similar to that of wild-type GP64 was detected (Fig. 6C), which indicated that this mutation did not alter GP64 secretion and fusion activity. Surprisingly, SP^Δn^GP64^A36G^ was detected on the cytomembrane and presented good fusion activity (Fig. 6C), which indicated that the uncleaved hydrophobic helix formed by the h-region is sufficient for protein secretion in BmN cells. Furthermore, mutated SPs were linked with Luciferase for quantitative analysis. As shown in Fig. 6C, in comparison with SP-Luc, SP^A36G^-Luc did not show any significant differences; a significant difference was detected in the SP^Δn-^Luc mutation, and the secretion rate of SP^ΔnA36G^-Luc was increased by 2.8-fold compared with SP^Δn-^Luc (Fig. 6C, left panel); however, the secretion rate of SP^ΔnA36G^-Luc was lower than that of SP-Luc.

### Helix disruption abolished protein secretion.

**FIG 7:**
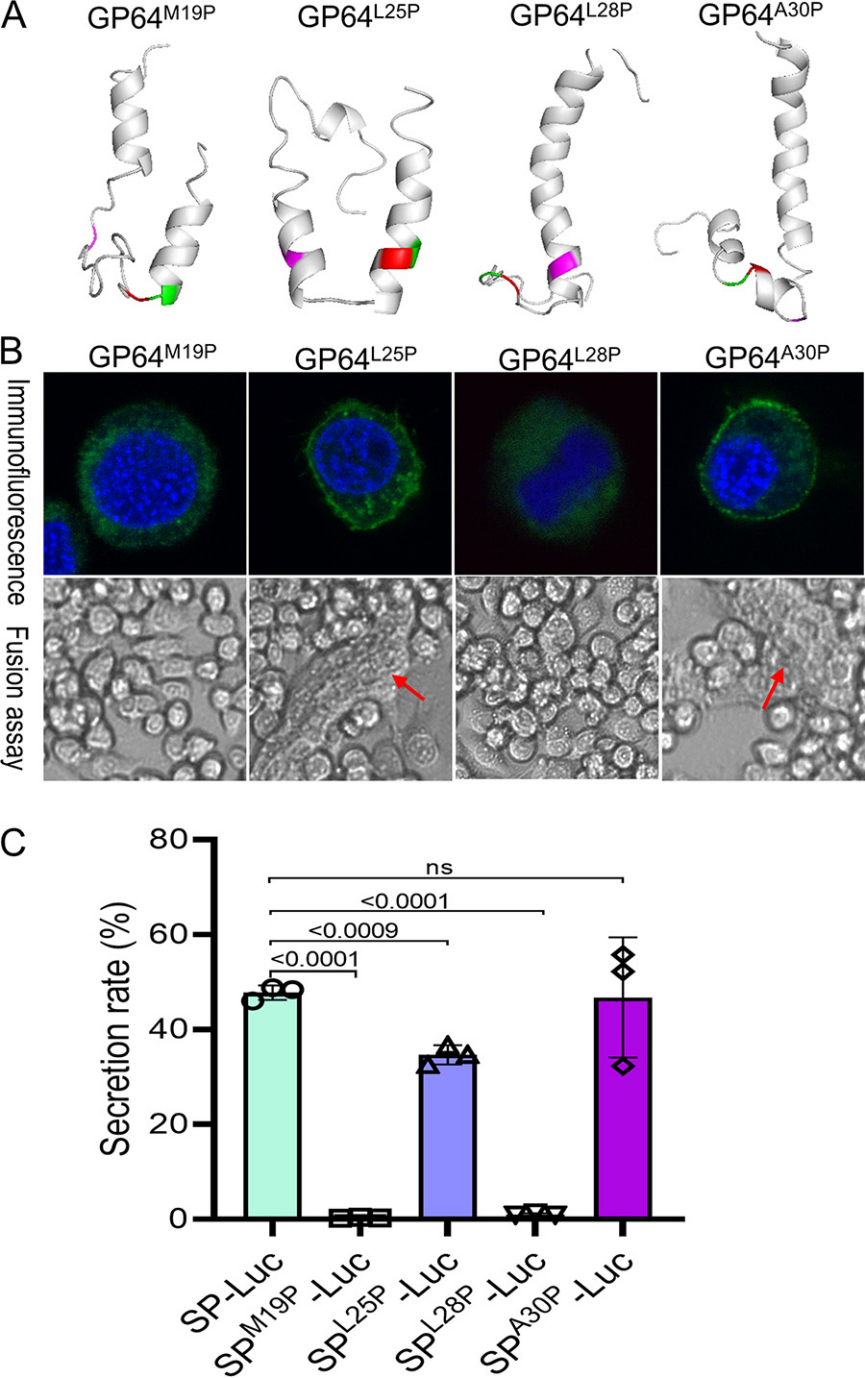
Helix breaking abolished protein secretion. (A) Prediction of helix breaking mutants of GP64. Four random residues in the helix of SP were mutated to proline to generate pIZ/V5-gp64^M19P^, pIZ/V5-gp64^L25P^, pIZ/V5-gp64^L28P^, or pIZ/V5-gp64^A30P^, and the mutant structure was predicted by Phyre2. The SPase cleavage site is shown in red and green, and the mutated prolines are shown in magenta. (B) Localization and membrane fusion assay of GP64 helix breaking mutants. BmN cells were transfected with pIZ/V5-gp64^M19P^, pIZ/V5-gp64^L25P^, pIZ/V5-gp64^L28P^, or pIZ/V5-gp64^A30P^, and then, the cells were subjected to immunofluorescence and fusion assays as described in B. (C) Secretion assay of SP with helix breaking mutations. Mutated SPs were amplified by PCR and inserted into pIZ/V5-Luc to generate pIZ/V5-SP^M19P^-Luc, pIZ/V5-SP^L25P^-Luc, pIZ/V5-SP^L28P^-Luc, and pIZ/V5-SP^A30P^-Luc. Then, these vectors and the control vectors pIZ/V5-SP-Luc were transfected into BmN cells, and the luciferase secretion rate and significance were analyzed at 72 h.p.t.

The helix structure is hardly accessible for proteinase binding, and we next verified this by helix-breaking analysis. Four residues in the helix were substituted with proline, which breaks the helix structure efficiently, and the secondary structure of mutations was predicted by Phyre2 (Fig. 7A). The GP64^M19P^ mutation disrupted the long helix into two helixes, which were linked by a loose loop structure, and the cleavage site was exposed in the loop. GP64^L25P^ disrupted the helix into three helixes, while this mutation did not alter the cleavage site location. GP64^L28P^ did not disrupt the long helix; instead, it shortened the helix, and the cleavage site was exposed to a loose loop region. The GP64^A30P^ mutation disrupted the helix; however, two residues of the cleavage site were localized in the helix.

Then, the location of the mutated GP64 was checked in BmN cells. As expected, GP64^M19P^ and GP64^L28P^ could not be transported to the cytomembrane (Fig. 7B, upper panel), and no fusion activity was detected in BmN cells (Fig. 7B, lower panel). GP64^L25P^ and GP64^A30P^ were still located on the membrane because the cleavage site was covered (Fig. 7B, upper panel), and good fusion activity was detected in BmN cells (Fig. 7B, lower panel). Furthermore, the role of the SP helix was also examined using quantitative analysis. The mutated SP was linked to *luciferase*, and the secretion rate of luciferase was compared. The M19P and L28P mutations abolished luciferase secretion, and the L25P mutation reduced the secretion rate, while the A30P mutant showed no difference from the SP-Luc control (Fig. 7C). These results are in agreement with the GP64 mutation assay, thus suggesting that the longer helix structure of SP is crucial for protein secretion.

## DISCUSSION

**FIG 8:**
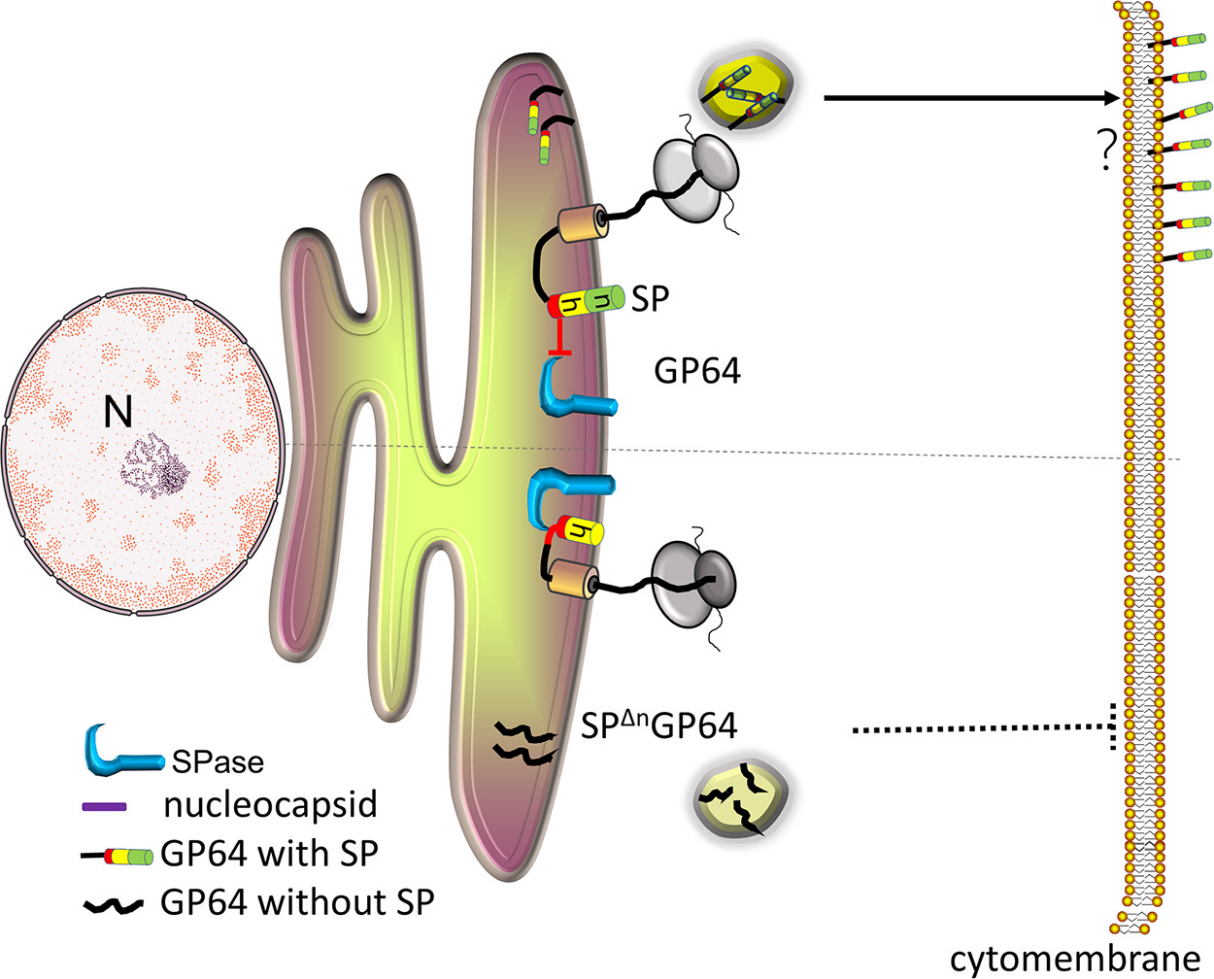
Model of BmNPV GP64 SP escaping cleavage to contribute to protein targeting in BmN cells. GP64 with an n-region of SP is translocated to the ER, where the helix structure covers the cleavage site and thus blocks cleavage from SPase, and then uncleaved SP mediates GP64 targeting to the cytomembrane (black arrow). Without the n-region, the cleavage site of SP was exposed in the loose structure, which was recognized and cleaved by SPase to produce SP^Δn^GP64 without SP; however, without the aid of SP, SP^Δn^GP64 could not be transported to the cytomembrane (black dotted line).

In this study, we revealed that BmNPV GP64 contains an uncleaved SP. To the best of our knowledge, this is the first report that a type I MFP retains its SP in the mature virion, although it contains an SPase cleavage site; therefore, we hypothesized that type I IMPs have two subtypes: type Ia IMPs possess a cleavable SP, while BmNPV GP64 belongs to type Ib IMPs, which possess an uncleaved SP. Based on our study, we proposed a model of GP64 escaping the cleavage of SPase (Fig. 8). Translation from the first initiation site of BmNPV GP64 encoded an n-region of SP, and the existence of the n-region extended the transmembrane helix, which covered the cleavage site and thus blocked cleavage from SPase. Then, GP64 conferred secretion ability across the cytomembrane in an unclear pathway (Fig. 8, black arrow). Meanwhile, translation from the second initiation site produced SP^Δn^GP64, and the absence of the n-region caused cleavage site exposure in a loose structure region. SP was then recognized and cleaved by SPase; thus, SP^Δn^GP64 cannot be transported to the cytomembrane (Fig. 8, black dotted line).

BmNPV GP64 escapes cleavage by its secondary structure around the SP, for the helical structure is hardly accessible for proteolysis (32). The same escape mechanism was reported in HIV GP160 (19), and the cleavage of GP160 SP was temporarily blocked until the protein finished modification. In other studies, the flanking sequence of the helix affects protein processing (31); in particular, positively charged residues are important for protein translocation and secretion (9, 33–35). The charge distribution of the GP64 SP correlates well with this “positive inside” rule (36); however, the mutagenesis assay did not alter protein localization, including multiple positively charged residue mutations. This may be caused by the protein's molecular weight; generally, small secretory proteins (<160 amino acids) depend more on the n-region positive charge than larger proteins (37).

Type I IMPs are secreted and anchored on the cytomembrane by the C-terminal TMD; however, BmNPV GP64 targeting on the cytomembrane requires the assistance of an uncleaved SP. We revealed that GP64 SP was required for Ebola virus glycoprotein cytomembrane localization in BmN cells but not for protein processing and glycosylation modification (38). Furthermore, both GP64 or SP^Δn^GP64 and their mutants were expressed and incorporated into virions to generate BVs with good infectivity (27), and these proteins were glycosylated in the secretion process. These results implied that proteins were transported into the Golgi apparatus. Thus, we inferred that SP-mediated GP64 transportation occurred from the Golgi apparatus to the cytomembrane. There are multiple protein trafficking pathways from the Golgi to the cytomembrane (39), including several vesicle transportation pathways. IMPs spanning the phospholipid bilayer need an α-helix of 3.75 nm in length (40), while we found that an approximately 2.3-nm helix can mediate SP^Δn^GP64^A36G^ cytomembrane localization; this length seems more suitable for single membrane insertion. Therefore, the transmembrane helix of SP may mediate GP64 insertion into a single membrane organelle to facilitate cytomembrane translocation; however, which pathway is involved in GP64 transportation is still unclear.

GP64 has been widely studied in past years, and clearer information has been obtained (41–44). However, as the key MFP of group I alphabaculovirus, the subtle investigation of the GP64 n-terminus is important for the understanding of GP64 characteristics because alternative initiation sites generate functionally distinct products (24). For AcMNPV GP64, regardless of which translation initiation site was used, both SPs of GP64 were removed, which is in line with the prediction result of the AcMNPV C6 and E2 strains (Fig. 1B). Similarly, our study is in alignment with the SP prediction of BmNPV GP64, which may be determined by the composition of SP. In another study, the BmNPV GP64 SP was used for fusion expression of human epidermal growth factor (hEGF, 53 amino acids); unexpectedly, a 12 kDa fusion product in BmN cells and silkworm was produced (45); moreover, its expression profile in larvae was similar to that of the luciferase secretion profile. These studies indicated that SP retention occurred not only in *B. mori* cells (BmN and Bm5) but also in larvae. BmNPV GP64 SP showed distinct functions from those of AcMNPV GP64, which is required not only for proteins to enter the secretory pathway but also for proteins to pass through the cytomembrane.

Longer SPs of viral proteins have diverse roles (20, 21, 46–50) and influence the topology of the mature prion protein (51, 52). Aligning with the finding that alternative translation generated functionally distinct isoform proteins (24), we revealed that SP-retained GP64 possessed a special dependence on cholesterol and strong virulence. BmNPV infection depends on host cholesterol and lipids (53–56), and the existence of GP64 SP contributes to the understanding of cholesterol dependence. Comparing one cholesterol-recognition amino acid consensus (CRAC) is required for virus with an SP-cleaved GP64, the same dependence mode with that of AcMNPV GP64, two CRAC motifs were required for BmNPV GP64; more importantly, this uncleaved SP offered BmNPV significantly higher BV production and virulence (27). This subtle variation is caused by SP retention, which is a key difference between AcMNPV and BmNPV GP64. GP64 is a new MFP acquired by group I alphabaculovirus in evolution (57). *B. mori*, the natural host for BmNPV, lost tree-climbing abilities during the domestication process and is dependent on humans for survival (58). SP retention may facilitate GP64 adaptation to *B. mori* cells or the receptor structure; thus, BmNPV can propagate better in silkworms. Therefore, uncleaved SP may be the coevolutionary result of host–virus interactions. Other baculoviruses, such as BomaNPV and RoMNPV, share even lower cleavage probability values of SP. RoMNPV is a variant of AcMNPV but is significantly more virulent against several pests (59); thus, the study of these viruses GP64 will enrich the SP function in virus infection.

In conclusion, we revealed that BmNPV GP64 retained its SP in the mature virion, which determined BmNPV virulence and other characteristics. We supposed that SP is a noteworthy study object given it alters protein function. Most SPs of the viral membrane protein were predicted, but only a few SP proteins were experimentally confirmed. Our results suggest that SP plays a vital role in protein translocation across the cytomembrane in BmN cells; therefore, it is necessary to continue the investigation to elucidate the function of SP in protein secretion.

## MATERIALS AND METHODS

### Cells and transfection.

BmN and Bm5 were cultured at 27°C in TC-100 insect medium (Applichem, Germany) supplemented with 10% fetal bovine serum (FBS, Gibco, USA), and Sf9 cells were cultured at 27°C in SF900II SFM (Thermo Fisher Scientific, Waltham, MA, USA) medium using standard techniques. BmN, Bm5, or Sf9 cells were in confocal dishes, 6-well plates, or 24-well plates (NEST Biotechnology, China) for overnight culture. Then, the cells were transfected with 0.8 μg, 4 μg, or 0.8 μg DNA of transient expression vectors by H4000 (Engreen Biosystem, Beijing, China) according to the manufacturer’s instructions, respectively.

### Bioinformatics analysis.

The SignalP server (https://services.healthtech.dtu.dk/service.php?SignalP-5.0) was used to predict the SP probabilities. JPred (http://www.compbio.dundee.ac.uk/jpred/) and Phyre2 (www.sbg.bio.ic.ac.uk/phyre2/) were used for protein secondary structure prediction. The helix length was measured by PyMOL (DeLano Scientific LLC).

### Construction of virus with Myc-tagged GP64.

The c-Myc tag was introduced into positions 18 and 19 of gp64 SP by overlap PCR. Briefly, using pIZ-V5-gp64 (60) as the template, two primers (Table S1) were used to amplify Myc-tagged gp64 by a high-fidelity enzyme (Miozyme, Shanghai, China). The product was inserted into pFBD-egfp (60) and then transposed into BmBacmid (61) to generate the recombinant bacmid BmBac-Myc-gp64. 4 μg bacmid DNA was mixed with H4000 and then transfected into BmN cells, the recombinant BV in the supernatant was collected at 120 h.p.t.

### Purification of BVs and Western blot analysis.

BmN cells were infected with the recombinant virus at a multiplicity of infection (MOI) of 1 TCID_50_ unit per cell. After 4 days, the cell culture supernatant was collected and purified as previously described (62). The purified BV pellet was resuspended in 100 μL of TE buffer for Western blotting as described (63). Myc monoclonal antibodies were purchased from Sangon Company (Shanghai, China), and GP64 and VP39 polyclonal antibodies were obtained from Manli Wang.

### BV envelope protein separation and MS analysis.

The envelope of purified BV was separated as described (64). Briefly, BmN cells were infected with BmBac-eGFP at an MOI of 5, and the supernatants were collected by centrifugation at 2,000 g for 10 min to remove the cell debris at 72 h.p.i., the supernatants were filtered through a 0.45-μm filter (Millipore), layered onto sucrose layers containing 2 mL of 60% sucrose and 5 mL of 25% sucrose in TE, and centrifuged for 120 min (Beckman SW40 rotor at 20,000 rpm at 4°C). The BV band was collected, diluted twice, and centrifuged for 90 min at 4°C (Beckman SW40, 24,000 rpm). The virus pellet was resuspended in 200 mL 0.1×TE, and then BVs were incubated in 1.0% N-P40 and 10 mM Tris, pH 8.5, at room temperature for 30 min with gentle agitation. The solution was then layered onto a 5-mL 30% (vol/vol) glycerol/10 mM Tris (pH 8.5) cushion and centrifuged at 150,000 g for 60 min at 4°C. The envelope fraction was recovered from the top of the gradient acetone precipitate and concentrated by centrifugation (4,000 × *g*, 30 min), and the pellet was dissolved in 10 mM Tris (pH 7.4) for SDS–PAGE separation and MS analysis (Hoogen Biotech company, Shanghai, China).

### GP64-Flag expression and purification in Sf9 cells.

*gp64* without a TMD (1 to 499 residues) was amplified by the primers gp64Fex and gp64FlagR, the PCR product was introduced into pFBD-egfp by StuI and PstI, the generated vector pFBD-egfp-gp64-Flag was transposed into the AcMNPV bacmid (Thermo Fisher Scientific, MA, USA), the recombinant bacmid was transfected into Sf9 cells to generate P0 and P1 viruses, and P1 virus was used to infect cells in a rolling bottle (NEST Biotechnology, China) at an MOI of 0.1. The supernatant was passed through a Flag affinity column (SABC, China) at a speed of 1 mL/min, and then the column was washed with PBS to remove unbound protein. The affinity column was incubated with glycine-HCl (pH 3.0), and the elution was collected at 1 mL per fraction, which contained 50 μL of neutralizing buffer (1 M Tris, pH 8.0). The purified GP64-Flag elution was checked by SDS–PAGE.

### N-terminal sequencing of GP64-Flag.

The GP64-Flag elution was condensed and subsequently separated by SDS–PAGE. The protein was then transferred to PVDF membranes for N-terminal sequencing (Applied Protein Technology, Shanghai, China). The peptides were blasted against GP64 of BmNPV in the database (NC_001962).

### Transient expression vector construction.

GP64 transient expression vectors were constructed by overlap PCR. pIZ/V5-gp64 (60) served as a template; *SP^Δn^gp64*, *SP^Δh-c^gp64*, *gp64^M19P^*, *gp64^L25P^*, *gp64^L28P^*, *gp64^A30P^*, *gp64^A36G^*, and *SP^Δn^gp64^A36G^* were amplified by the overlapping PCR method using the primer pairs shown in Table S1, and the products were inserted into the pIZ/V5 vector by EcoR I and XbaI. All constructs were verified by DNA sequencing. For quantitative analysis of SP secretion efficiency, the n-region, h-c region, SP^A36G^, SP^M19P^, SP^L25P^, SP^L28P^, SP^A30P^, and SP^ΔnA36G^ were inserted into pIZ/V5-Luc (26) to generate pIZ/V5-SP^Δn^-Luc, pIZ/V5-SP^Δh-c^-Luc, pIZ/V5-SP^A36G^-Luc, pIZ/V5-SP^M19P^-Luc, pIZ/V5-SP^L25P^-Luc, pIZ/V5-SP^L28P^-Luc, pIZ/V5-SP^A30P^-Luc, and pIZ/V5-SP^ΔnA36G^-Luc. SP alanine mutagenesis fragments and SP truncated fragments were synthesized (Sangon, Shanghai, China) as shown in Table S1 and were inserted into pIZ/V5-eGFP-TMD or pIZ/V5-Luc to generate transient expression vectors. All constructs were verified by DNA sequencing and then transfected into cells.

### Immunofluorescent assay.

BmN and Sf9 cells were transfected with GP64 or GP64 mutants and then washed with serum-free medium three times at 72 h.p.t. The cells were fixed with 4% paraformaldehyde, and an immunofluorescence assay was conducted with a GP64 antibody and FITC-labeled secondary antibody as previously described (60). The nucleus was stained with Hoechst 33258.

### Fusion assay.

BmN or Sf9 cells were preseeded in six-well plates, and then the cells were transfected with GP64 and GP64 mutant DNA. The cells were then incubated with low-pH medium (pH 4.5) at 72 h.p.t. to trigger membrane fusion for 5 min, and then the cells were cultured with normal TC100 media for 4 h. The nucleus was stained with Hoechst 33258 and imaged as previously described (60).

### Live-cell imaging.

BmN, Bm5, and Sf9 cells were transfected with transient expression vectors of eGFP led by different SPs. The cells were stained with rhodamine B chloride (R18, Sigma–Aldrich, MS, USA) and Hoechst to label the plasma membrane and nucleus, respectively, at 72 h.p.t., and fluorescence was imaged by confocal microscopy (Leica SP8).

### Luciferase activity assay.

BmN or Sf9 cells in 24-well plates were transfected with 0.8 μg of luciferase expression vector DNA and cultured normally. Then, the medium and cells were harvested and lysed at the indicated time points (26). The secretion rate was calculated by the relative luciferase unit (RLU) in the media averaged by the RLU of total media and cells. Three independent biological replicates were performed, and *P* values were generated with a two-tailed *P* value from the *t* test in GraphPad Prism 8.
