# Supplementary material for: The Bombyx mori Nucleopolyhedrovirus GP64 Retains the Transmembrane Helix of Signal Peptide to Contribute to Secretion across the Cytomembrane
Source: Microbiol Spectr. 2022 Aug 8;10(4):e01913-22. doi: 10.1128/spectrum.01913-22 (PMC9430547; doi:10.1128/spectrum.01913-22)
Supplement: Supplemental file 1 — Supplemental material. Download spectrum.01913-22-s0001.pdf, PDF file, 0.1 MB [file spectrum.01913-22-s0001.pdf]

Table S1. Primers used in this study

**Name**	**Sequence (5′-3′)**
Progp64-F	CGCGAATTCGACAGATATTTAAATAAACCAAAC
Progp64-R	GCGTCTAGATTAATATTGTCTACTATTACGGTT
Myc-gp64-F	*GAGCAGAAGCTGATCTCGGAGGAGGATCTG*ATGGTAGGCGCTATTGTTTT (c-Myc sequence shown in italic)
Myc-gp64-R	*CAGATCCTCCTCCGAGATCAGCTTCTGCTC*CTCGCTTGTGTGTTT (c-Myc sequence shown in italic)
gp64Fex	GGCAGGCCTATGCTACTAGTAAATCAGTCATAC
gp64FlagR	GCCCTGCAG*CTTATCGTCGTCATCCTTGTAATC*GAACGAAGTCAATTTGGCGGC (Flag-tag shown in italic)
SP$^{\Delta n}$gp64R	AATAGCGCCTACCATTGAGGCATCTTATATACCCGA
SP$^{\Delta n}$gp64F	ATATAAGATGCCTCAATGGTAGGCGCTATTGTTTTA
SP$^{\Delta h\text{-}c}$gp64R	TGCGTTGCAGTGCTCCTCGCTTGTGTGTTTCTTATC
SP$^{\Delta h\text{-}c}$gp64F	AAACACACAAGCGAGGAGCACTGCAACGCACAAATG
GP64M19PF	GATAAGAAACACACAAGCGAGCCGGTAGGCGCTATTGTTTTATAC
GP64M19PR	GTATAAAACAATAGCGCCTACCGGCTCGCTTGTGTGTTTCTTATC
GP64L25PF	AGATGGTAGGCGCTATTGTTCCATACGTGCTTTTGGCGGC
GP64L25PR	GCCGCCAAAAGCACGTATGGAACAATAGCGCCTACCATCT
GP64L28PF	CGCTATTGTTTTATACGTGCCTTTGGCGGCGCATTCTGC
GP64L28PR	GCAGAATGCGCCGCCAAAGGCACGTATAAAACAATAGCG
GP64A30PF	CTATTGTTTTATACGTGCTTTTGCCGGCGCATTCTGCCTTTG
GP64A30PR	CAAAGGCAGAATGCGCCGGCAAAAGCACGTATAAAACAATAG
GP64A36GF	GCATTCTGCCTTTGGGGCGGAGCACTGCAA
GP64A36GR	TTGCAGTGCTCCGCCCCAAAGGCAGAATGC
SPF	GCGGGTACCATGCTACTAGTAAATCAGTCAAC
SPA36GR	GCGGAGCTCCCCAAAGGCAGAATGCGCCG
SPL2A	CGGGGTACCATGGCACTAGTAAATCAGTCATACCAAGGCTTCGATAAGAAACACACAAGCGAGATGGTAGGCGCTATTGTTTTATACGTGCTTTTGGCGGCGCATTCTGCCTTTGCGGCGGAGCTCGCG
SPL3A	CGGGGTACCATGCTAGCAGTAAATCAGTCATACCAAGGCTTCGATAAGAAACACACAAGCGAGATGGTAGGCGCTATTGTTTTATACGTGCTTTTGGCGGCGCATTCTGCCTTTGCGGCGGAGCTCGCG
SPV4A	CGGGGTACCATGCTACTAGCAAATCAGTCATACCAAGGCTTCGATAAGAAACACACAAGCGAGATGGTAGGCGCTATTGTTTTATACGTGCTTTTGGCGGCGCATTCTGCCTTTGCGGCGGAGCTCGCG
SPN5A	CGGGGTACCATGCTACTAGTAGCACAGTCATACCAAGGCTTCGATAAGAAACACACAAGCGAGATGGTAGGCGCTATTGTTTTATACGTGCTTTTGGCGGCGCATTCTGCCTTTGCGGCGGAGCTCGCG
SPQ6A	CGGGGTACCATGCTACTAGTAAATGCGTCATACCAAGGCTTCGATAAGAAACACACAAGCGAGATGGTAGGCGCTATTGTTTTATACGTG

	CTTTTGGCGGCGCATTCTGCCTTTGCGGCGGAGCTCGCG
	CGGGGTACCATGCTACTAGTAAATCAGGCATACCAAGGCTTCGATAAG
SPS7A	AAACACACAAGCGAGATGGTAGGCGCTATTGTTTTATACGTGCTTTTG
	GCGGCGCATTCTGCCTTTGCGGCGGAGCTCGCG
	CGGGGTACCATGCTACTAGTAAATCAGTCAGCACAAGGCTTCGATAAG
SPY8A	AAACACACAAGCGAGATGGTAGGCGCTATTGTTTTATACGTGCTTTTG
	GCGGCGCATTCTGCCTTTGCGGCGGAGCTCGCG
	CGGGGTACCATGCTACTAGTAAATCAGTCATACGCAGGCTTCGATAAG
SPQ9A	AAACACACAAGCGAGATGGTAGGCGCTATTGTTTTATACGTGCTTTTG
	GCGGCGCATTCTGCCTTTGCGGCGGAGCTCGCG
	CGGGGTACCATGCTACTAGTAAATCAGTCATACCAAGCCTTCGATAAG
SPG10A	AAACACACAAGCGAGATGGTAGGCGCTATTGTTTTATACGTGCTTTTG
	GCGGCGCATTCTGCCTTTGCGGCGGAGCTCGCG
	CGGGGTACCATGCTACTAGTAAATCAGTCATACCAAGGCGCCGATAAG
SPF11A	AAACACACAAGCGAGATGGTAGGCGCTATTGTTTTATACGTGCTTTTG
	GCGGCGCATTCTGCCTTTGCGGCGGAGCTCGCG
	CGGGGTACCATGCTACTAGTAAATCAGTCATACCAAGGCTTCGCTAAG
SPD12A	AAACACACAAGCGAGATGGTAGGCGCTATTGTTTTATACGTGCTTTTG
	GCGGCGCATTCTGCCTTTGCGGCGGAGCTCGCG
	CGGGGTACCATGCTACTAGTAAATCAGTCATACCAAGGCTTCGATGCT
SPK13A	AAACACACAAGCGAGATGGTAGGCGCTATTGTTTTATACGTGCTTTTG
	GCGGCGCATTCTGCCTTTGCGGCGGAGCTCGCG
	CGGGGTACCATGCTACTAGTAAATCAGTCATACCAAGGCTTCGATAAG
SPK14A	GCTCACACAAGCGAGATGGTAGGCGCTATTGTTTTATACGTGCTTTTG
	GCGGCGCATTCTGCCTTTGCGGCGGAGCTCGCG
	CGGGGTACCATGCTACTAGTAAATCAGTCATACCAAGGCTTCGATAAG
SPH15A	AAAGCCACAAGCGAGATGGTAGGCGCTATTGTTTTATACGTGCTTTTG
	GCGGCGCATTCTGCCTTTGCGGCGGAGCTCGCG
	CGGGGTACCATGCTACTAGTAAATCAGTCATACCAAGGCTTCGATAAG
SPT16A	AAACACGCAAGCGAGATGGTAGGCGCTATTGTTTTATACGTGCTTTTG
	GCGGCGCATTCTGCCTTTGCGGCGGAGCTCGCG
	CGGGGTACCATGCTACTAGTAAATCAGTCATACCAAGGCTTCGATAAG
SPS17A	AAACACACAGCCGAGATGGTAGGCGCTATTGTTTTATACGTGCTTTTG
	GCGGCGCATTCTGCCTTTGCGGCGGAGCTCGCG
	CGGGGTACCATGCTACTAGTAAATCAGTCATACCAAGGCTTCGATAAG
SPE18A	AAACACACAAGCGCGATGGTAGGCGCTATTGTTTTATACGTGCTTTTG
	GCGGCGCATTCTGCCTTTGCGGCGGAGCTCGCG
	GAGCTCATGCTACAGTCATACCAAGGCTTCGATAAGAAACACACAAG
SP$^{\Delta}$3	CGAGATGGTAGGCGCTATTGTTTTATACGTGCTTTTGGCGGCGCATTCT
	GCCTTTGCGGCGGGATCC
	GAGCTCATGTACCAAGGCTTCGATAAGAAACACACAAGCGAGATGGT
SP$^{\Delta 6}$	AGGCGCTATTGTTTTATACGTGCTTTTGGCGGCGCATTCTGCCTTTGCG
	GCGGGATCC
SP$^{\Delta 9}$	GAGCTCATGTTCGATAAGAAACACACAAGCGAGATGGTAGGCGCTAT

	TGTTTTATACGTGCTTTTGGCGGCGCATTCTGCCTTTGCGGCGGGATCC
$SP^{\Delta 12}$	GAGCTCATGAAACACACAAGCGAGATGGTAGGCGCTATTGTTTTATAC<br>GTGCTTTTGGCGGCGCATTCTGCCTTTGCGGCGGGATCC
$SP^{\Delta 15}$	GAGCTCATGAGCGAGATGGTAGGCGCTATTGTTTTATACGTGCTTTTG<br>GCGGCGCATTCTGCCTTTGCGGCGGGATCC
$SP^{\Delta 16}$	GAGCTCAGCGAGATGGTAGGCGCTATTGTTTTATACGTGCTTTTGGCG<br>GCGCATTCTGCCTTTGCGGCGGGATCC
$SP^{\Delta 17}$	GAGCTCGAGATGGTAGGCGCTATTGTTTTATACGTGCTTTTGGCGGCG<br>CATTCTGCCTTTGCGGCGGGATCC
